# Disease and Patient Characteristics Contributing to Diagnostic Delays in Patients With Guillain-Barré Syndrome

**DOI:** 10.3389/fneur.2021.684847

**Published:** 2021-06-25

**Authors:** Chakrapani Pathikonda, Nakul Katyal, Naureen Narula, Raghav Govindarajan

**Affiliations:** ^1^Department of Neurology, University of Missouri, Columbia, MO, United States; ^2^Department of Pulmonology and Critical Care, Staten Island University Hospital, New York, NY, United States

**Keywords:** demyelination, diagnostic delays, polyneuropathy, immune mediated neuropathies, GBS

## Abstract

**Introduction:** Diagnosis of Guillain Barre syndrome (GBS) is often made clinically. Certain patient and disease characteristics can cause delays in diagnosis and management.

**Methods:** Observational retrospective study of forty-four patients diagnosed with GBS either clinically, cerebrospinal fluid analysis, and/or by electro-diagnostic criteria at a teaching hospital (University of Missouri Hospital) in Columbia, Mid-Missouri between 2011 and 2017.

**Results:** Patients with coexisting neurological conditions had statistically significant delay in diagnosis of GBS [Mean (SD); 13 ± 5 vs. 9.39 ± 4.7; *p* = 0.03]. Patients presenting with motor + symptoms (sensory and/or autonomic, in addition to motor), compared to those with only motor symptoms had statistically significant delay in diagnosis of GBS [Mean (SD); 11.90 ± 5 vs. 8.58 ± 4; *p* = 0.04].

**Discussion:** Presence of co-existing neurological conditions, and motor + symptoms can delay timely diagnosis and management of GBS.

## Introduction

Guillain-Barre Syndrome (GBS) is a heterogenous group of immune-mediated peripheral neuropathies with demyelinating and acute axonal degenerating pathologies (1). Classic presentation has symmetric limb weakness progressing over the course of days and absence of deep tendon reflexes on examination (1, 2). The diagnosis of GBS is often made clinically as sensitivities of objective findings in electrodiagnostic study and cerebrospinal fluid (CSF) analysis are low in the first 1–2 weeks of disease onset (2). Early supportive management, close monitoring of respiratory status, admission to intensive care units, and initiation of immunomodulatory therapy are key factors for favorable outcomes (1–3). GBS has a mortality rate of 3–13% therefore, prompt diagnosis and treatment is paramount (3, 4). First-contact physicians must pay close attention to the presenting clinical symptoms and temporality of symptom onset to consider GBS as a differential and ensure prompt diagnosis.

In this observational study, we retrospectively reviewed data of 44 patients diagnosed with GBS between 2011 and 2017 at a teaching hospital and analyzed patient and disease characteristics contributing to delay in diagnosis of GBS.

## Methods

This study was approved by the institutional review board of University of Missouri-Columbia. Informed consent was waived by the Institutional Review Board. IRB Approval number #2008796.

### Patient Selection Criteria

A retrospective chart review was conducted of data from patients diagnosed with GBS at the University Hospital (UH), Columbia, Missouri between 2011 and 2017. We identified patients diagnosed with GBS under ICD-9 code of 357.0. The inclusion criteria were further narrowed to include those with a documented diagnosis of Acute Demyelinating Inflammatory Polyradiculoneuropathy (AIDP), and variants of GBS including Acute Motor and Sensory Axonal Neuropathy (AMSAN), Acute Sensory Axonal Neuropathy (ASAN), Miller Fisher Syndrome, and Pharyngeal Cervical Brachial Variant and those that met the criteria for acute onset of progressive weakness in one or more limbs and diminished or absent tendon reflexes compared to the patient's baseline status. We included only newly diagnosed cases that presented during the study period. Patients who were initially suspected of having GBS but eventually diagnosed with a different condition, patients with incomplete documentation, and those with other significant medical conditions that complicated patients' stay and management in the hospital were excluded from the study.

**Figure 1:**
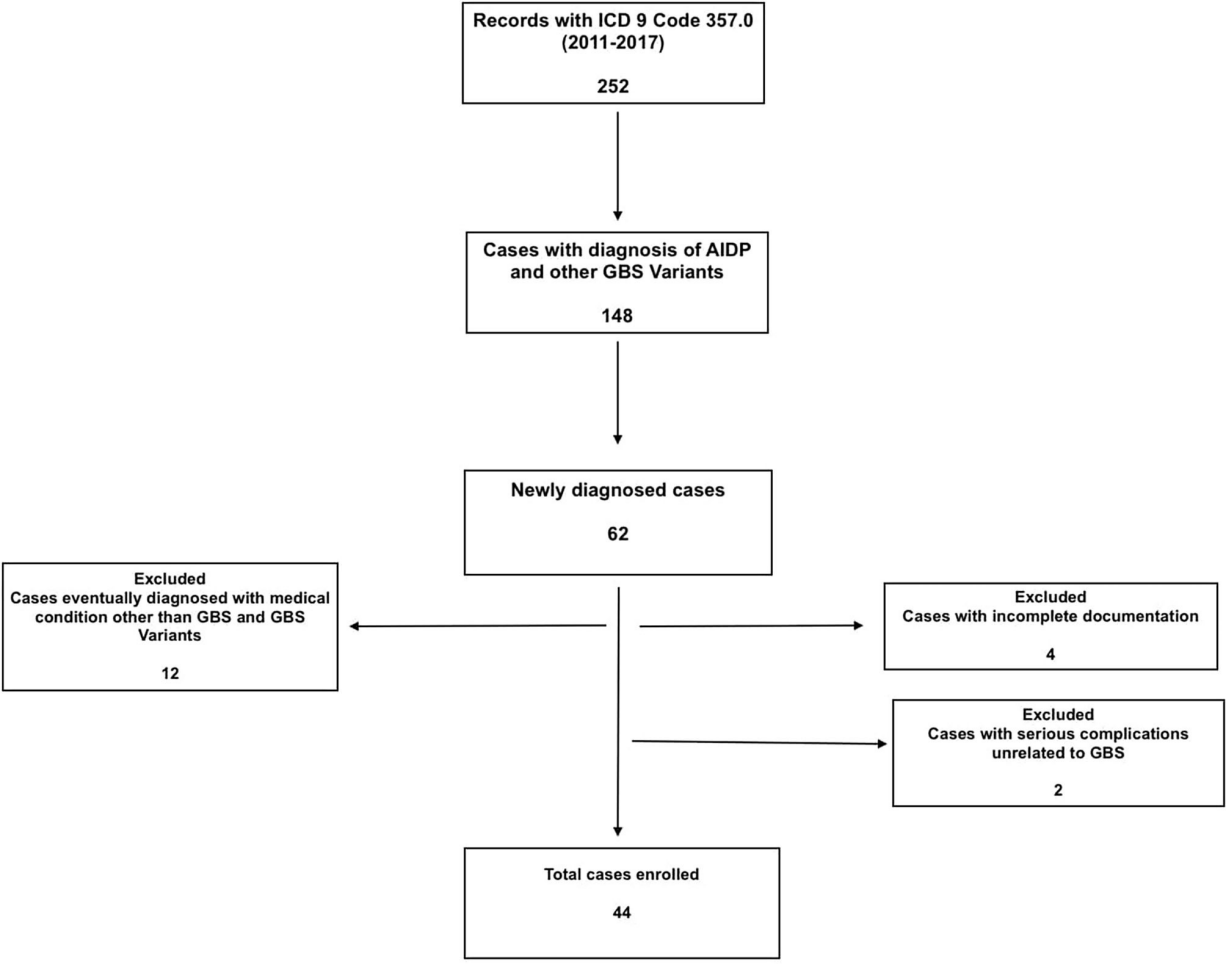
Consort diagram for patient identification.

A total of 252 patient charts were screened based on ICD-9 code, and 62 cases with GBS and GBS variants were identified. Twelve of the patients who were initially investigated for GBS, but eventually diagnosed with other medical conditions were excluded. Four cases were excluded due to incomplete documentation, mostly from outside hospitals where they presented initially. Two cases were excluded because their management was complicated by other serious conditions unrelated to GBS (complications associated with hemodialysis and hydrocephalus with shunt) (Figure 1).

### Demographic Characteristics

Demographic data including patient age, gender, race and location (urban vs. rural based on 2010 Census Urban and Rural Classification and Urban area criteria) were extracted from electronic medical records. If available, information from prior clinic and or hospital visits, indicating symptom onset and GBS diagnosis/suspicion was included for data analysis.

### Disease Characteristics

Disease characteristics included type of symptoms [motor vs. motor + (sensory and or autonomic, in addition to motor)], location of symptoms (lower limbs only vs. combination which includes lower limbs with upper limbs/facial/bulbar), pre-existing medical conditions, antecedent history of illness (within 3 weeks before symptoms onset; upper respiratory or gastrointestinal) or immunization or surgery, and severity of symptoms. Severity of symptoms at the time of admission was defined as (1) Mild: if patient had occasional limitation of function from baseline, (2) Moderate: if patient had significant and persistent limitation of function from baseline at home or occupation, needing admission to general floor of hospital, and (3) Severe: if patient had impaired swallowing or breathing status, limb fracture due to falls, requiring admission to Intensive Care Unit (ICU).

### CSF Analysis and Electrodiagnostic Studies

Electrodiagnostic findings consistent with GBS including an absent H reflex, sural sparing pattern, prolongation of onset latency, slowing of conduction velocity, and an abnormal F wave were considered diagnostic (1). Similarly, CSF study findings consistent with albumin-cytologic dissociation (elevated CSF protein content > 50 mg/dL and WBC cell count < 5/dl) were considered diagnostic (5).

### Data Analysis

Mean time to diagnosis was compared between multiple variables including demographics, disease characteristics, number of days since symptom onset, diagnosis/suspicion of GBS within 24 h of admission, neurology consultation within 24 h of admission, EMG and CSF analysis.

### Statistical Analysis

Continuous and categorical data were summarized with descriptive statistics including means with standard deviations. Only data with normal distribution was used to calculate means with standard deviation. Categorical analyses were performed using Graph Pad 7.0 (La Jolla, CA, USA). Mean and standard deviations were compared between different groups. Ninety five percent confidence intervals (CI), and differences were considered statistically significant at *P* < 0.05.

## Results

### Demographic Analysis

A total of 44 patients who met the study criteria were included in the analysis. Twenty seven (61%) were males, 17 (39%) were females. The mean (standard deviation [SD]) age was 48 years (±14.9 years). 20 patients (45%) lived in rural locations and 24 (55%) in urban locations. All 20 patients (100%) from rural locations visited an outside provider (primary care clinic or community hospital) before presenting to our hospital. Only 4 out of 24 (17%) of urban patients visited an outside provider (clinic or hospital) before presenting to our hospital.

### Disease Characteristics

Out of a total of 44 patients, 33 patients were diagnosed with AIDP, 1 with AMAN, 6 with AMSAN, 3 with Miller Fischer, 1 with Pharyngeal Cervical Brachial Variant of GBS.12 out of 44 patients (27%) had neurological symptoms limited to motor modality, while the remaining 32 patients (63%) had motor + symptoms involving motor and/ or other modalities including sensory and autonomic. At the time of presentation to the emergency department, 30 patients (68%) had symptoms in other locations of the body (upper extremities, facial/ bulbar) in addition to the lower limbs, while 14 patients (32%) had symptoms limited to lower limbs. Twelve patients (27%) had coexisting neurological conditions including lumbar spondylolysis, cervical stenosis, back pain, previous lumbar disc herniation status post laminectomy, peripheral neuropathy, stroke, spinal stenosis, and seizures. Thirty two patients (73%) had either cardiovascular or endocrine co-existing conditions such as hypertension, diabetes mellitus and thyroid disorders. Antecedent history of upper respiratory infection or gastrointestinal infection or immunization or surgery was present in a total of 26 patients (59%), remaining 18 patients (41%) did not have any preceding history of recent infections. Twenty six patients (59%) had moderate symptoms at time of presentation, while 17 patients (38%) had severe symptoms. Only 1 patient (<1%) had mild symptoms at time of presentation.

### CSF Analysis and Electro Diagnostic Studies

Prior to initiating treatment for GBS, electrodiagnostic study results were available in 73% of patients (*n* = 32) and CSF analysis results were available in 59% of patients (*n* = 26).

In 5 patients (11%), both results from electrodiagnostic studies and CSF analysis results were diagnostic. In 16 patients (82%), either one of electrodiagnostic studies or CSF analysis results were diagnostic. In 3 patients (7%) both electrodiagnostic studies and/or CSF results were inconclusive. Table 1 describes the clinical characteristics of patients with GBS.

**Table 1:** Clinical characteristics of patients with Guillain-Barre syndrome.

**Variables**		**Number of patients**	**Percentages (%)**
**Demographics**	Female/Male	17/27	39/61
	Rural/Urban	20/24	45/55
Co-existing conditions/Past medical diagnoses	Neurological^*^	12	27
	Other^**^	32	73
**Visits to outside clinic or hospital prior to UH**			
Number of visits	≧1	34	77
	None	10	23
GBS diagnosed/suspected at first encounter	Yes	12	27
	No	32	73
Antecedent history	None	18	41
	URI	18	41
	GI	5	11
	Immunization	2	1
	Surgery	1	<1
**Symptoms**			
Affected body locations	Lower limbs only	14	32
	Combination	30	68
Modality affected	Motor only	12	27
	Motor+	32	73
Severity	Mild	18	40
	Moderate	23	52
	Severe	3	7
Electrodiagnostic study prior to treatment	No	12	27
	Yes	32	73
CSF study prior to treatment	No	18	41
	Yes:	26	59
Diagnosis confirmation by objective findings in electrodiagnostic or CSF studies	Both	5	11
	Either, not both	36	82
	Neither	3	7
GBS diagnosis in <24 h of UH visit	Yes	32	73
	No	12	27
Neurology consultation	<24 h	38	86
	>24 h	6	14

*UH, University of Missouri Hospital; URI, Upper Respiratory Infection; GBS, Guillain-Barre Syndrome; GI, Gastrointestinal Infection; CSF, Cerebrospinal Fluid*.

* *Examples include spondylosis, spinal stenosis, lumbar disc herniation*.

** *Examples include hypothyroidism, hypertension, diabetes mellitus, irritable bowel syndrome*.

### Statistical Analysis

GBS diagnosis was correctly suspected or diagnosed in 73% of cases (*n* = 32) within 24 h of presentation, while in 27% of cases (*n* = 12), the diagnosis was after 24 h of presentation to the emergency department. Neurology consultation was requested within 24 h of visit in majority of cases (86%, *n* = 38). On comparative statistical analysis of mean time to diagnosis across all identified variables, co-existing neurologic conditions and presence of motor + symptoms were associated with statistically significant delay in diagnosis of GBS (*p* < 0.05).

Table 2 describes the comparative analysis of mean time to diagnosis across all identified variables.

**Table 2:** Comparative analysis of mean time to diagnosis across all identified variables.

		**Number of patients**	**Time to diagnosis: Mean ± SD. (days)**	***P***
Age	<50 Years	21	11 ± 4.16	0.83
	>50 years	23	10.69 ± 5.55	
Gender	Female	17	9.35 ± 5.07	0.11
	Male	27	11.77 ± 4.61	
Demographics	Urban	24	11.85 ± 4.51	0.79
	Rural	20	11.43 ± 4.53	
Disease type	AIDP	33	10.57 ± 4.91	0.53
	GBS Variants^*^	11	11.63 ± 4.94	
Outside provider visits prior to UH	Clinic/hospital visits	34	11.55 ± 4.9	0.07
	None	10	8.4 ± 4.2	
Clinical severity at admission time	Mild	18	12.27 ± 5.1	0.1
	Moderate/Severe	26	9.84 ± 4.6	
Co-existing conditions	Neurological	12	13 ± 5.0	**0.03**
	Other	32	9.39 ± 4.7	
Antecedent history	Present	26	10.69 ± 5.5	0.81
	Absent	18	11.05 ± 4.3	
Symptom location	Lower limb	14	9.5 ± 5.2	0.22
	Mixed	30	11.46 ± 4.8	
Symptom modality	Motor	12	8.58 ± 4.0	**0.04**
	Combination	32	11.90 ± 5.0	
GBS diagnosis at UH	<24 h	32	11.34 ± 5.4	0.27
	>24 h	12	9.5 ± 3.0	
GBS diagnosis at 1st encounter	Yes	11	8.63 ± 4.58	0.09
	No	33	11.57 ± 4.97	
Neurology consultation	<24 h	38	11 ± 5.1	0.59
	>24 h	6	9.83 ± 3.6	

*UH, University of Missouri Hospital; SD, Standard deviation; GBS, Guillain-Barre Syndrome*.

GBS Variants^*^: Acute Motor and Sensory Axonal Neuropathy (AMSAN), Acute Sensory Axonal Neuropathy (ASAN), Miller Fisher Syndrome, and Pharyngeal Cervical Brachial Variant. Bold values are statistically significant.

Ten out of 12 (84%) patients with motor symptoms alone were discharged to inpatient rehab and remaining 2 (16%) patients were discharged home. In comparison, in patients with motor + symptoms, 20 out of 32 (63%) patients with motor + symptoms were discharged home and 12 out of 32 (37%) patients were discharged to inpatient rehab.

Eight out of 12 (67%) patients with preexisting neurological symptoms were discharged home, remaining 4 (33%) patients were discharged to inpatient Rehab. In comparison, 13 out of 32 (40%) patients with other co-existing conditions were discharged home and 19 out of 32 (60%) patients were discharged to inpatient rehab.

## Discussion

In our study, co-existing neurologic conditions and presence of motor + symptoms (sensory and/or autonomic in addition to motor) at time of initial presentation were associated with delay in the diagnosis of GBS. In the majority of the delayed cases, the working hypotheses were either intracranial/spinal cord pathologies or peripheral nerve pathologies. Transverse myelitis, cord compression, and radiculopathies were considered the differentials among intracranial/spinal cord pathologies. Heavy metal intoxication, tick paralysis and metabolic disturbances (hypoglycemia, hypokalemia, hypophosphatemia) were considered the most common working diagnoses among peripheral nerve pathologies. These differential diagnoses were consistent with the spectrum of commonly reported differential diagnosis with GBS reported in previous studies (6).

Previous studies have described challenges with diagnosis of GBS in emergency room settings (7, 8). In a study by Dubey et al. GBS diagnosis was not suspected during initial emergency department visits in nearly 50% of patients (8). Similarly, in study by McGillicuddy et al. 65% of patients (*n* = 13) required more than one evaluation before GBS was diagnosed (9). A meticulous approach, due diligence to specific disease patterns, onset duration, progression, and antecedent history can help delineate coexisting conditions from GBS (10). Our study supports these findings and extends to include primary care clinics in rural settings. Possible solutions to avoid misdiagnosis include physician education about neurological emergencies that encourage detailed history-taking and systematic physical examination, real-time neurology consultation and clear communication with patients and consultant physicians (11).

Another significant finding in our study was the association between neurological symptom modality and time to diagnosis. In our study, majority of patients (73%, *n* = 32) had motor + symptoms, whereas motor symptoms alone were present in 27% of patients (*n* = 12). This finding was consistent with previous reports by Fokke et al. where sensory deficits were seen in 67% of patients (322 out of 480) in addition to limb weakness (12). This underscores the importance of considering co-existing non-motor symptoms in clinical diagnosis of GBS.

About 60% of patients have infectious symptoms, in the 3 weeks before the onset of weakness, resulting predominantly from upper respiratory tract or gastrointestinal tract infections (13). One Japanese study in 2001 found that the most frequent antecedent symptoms in GBS were fever (52%), cough (48%), sore throat (39%), nasal discharge (30%), and diarrhea (27%) (13). Similarly, in our study population 41% of patients had preceding respiratory illness and 11% had GI symptoms prior to onset of weakness.

There are some limitations to our study. The findings of this retrospective study at a single institution may not be reflective of outcomes in other care settings. Some GBS patients may have been overlooked if the clinical diagnosis was coded incorrectly. Not all patient records from outside hospitals stated differential diagnosis, hence, it is possible that we may have undercounted the GBS suspicion at the outside facilities. Lastly, the small sample size and lack of control group limit the robustness of our findings.

## Conclusion

Presence of coexisting neurological conditions, and non-motor symptoms (in addition to motor symptoms) can delay timely diagnosis of GBS.

## Data Availability Statement

The original contributions presented in the study are included in the article/supplementary material, further inquiries can be directed to the corresponding authors.

## Ethics Statement

The studies involving human participants were reviewed and approved by University of Missouri IRB #2008796. Written informed consent from the participants' legal guardian/next of kin was not required to participate in this study in accordance with the national legislation and the institutional requirements.

## Author Contributions

All authors contributed equally to literature writing, review, and editing.

## Conflict of Interest

The authors declare that the research was conducted in the absence of any commercial or financial relationships that could be construed as a potential conflict of interest.
